# A global analysis of National Malaria Control Programme vector surveillance by elimination and control status in 2018

**DOI:** 10.1186/s12936-019-3041-2

**Published:** 2019-12-04

**Authors:** Thomas R. Burkot, Robert Farlow, Myo Min, Effie Espino, Abraham Mnzava, Tanya L. Russell

**Affiliations:** 1 Australian Institute of Tropical Health and Medicine, James Cook University, Cairns, Australia; 2 R. Farlow Consulting LLC, Burkeville, TX USA; 3 Asia-Pacific Malaria Elimination Network, Singapore, Singapore; 4 African Leaders’ Malaria Alliance, Dar es Salaam, Tanzania

**Keywords:** Malaria vector surveillance, Malaria control intervention access and use, Vector indicators, Malaria elimination

## Abstract

**Background:**

Maintaining the effectiveness of the currently recommended malaria vector control interventions while integrating new interventions will require monitoring key recommended indicators to identify threats to effectiveness including physiological and behavioural resistance to insecticides.

**Methods:**

Country metadata on vector surveillance and control activities was collected using an online survey by National Malaria Control Programmes or partner organization officials. Country and regional surveillance activities were analysed for alignment with indicators for priority vector surveillance objectives recommended by the World Health Organization. Surveillance activities were also compared for countries in the E2020 (eliminating countries) and countries with more intense transmission.

**Results:**

Significant differences in monitoring priority vector indicators between Africa and Asia-Pacific country programmes were found as well as differences between countries approaching elimination and those controlling malaria. Gaps were found between vector data collected and country management strategies (i.e., for insecticide resistance management and integrated vector control strategies) and for making programmatic decisions on surveillance and control using vector surveillance data.

**Conclusions:**

Significant opportunities exist for increasing vector data collection on priority indicators and using these data for national programmatic decisions for both proactive insecticide resistance management and enhancing vector control.

## Background

Global malaria cases decreased significantly from 2000 to 2015 with most of the reduction attributed to vector control with insecticide-treated nets (ITNs) and indoor residual spraying (IRS) [1, 2]. After a period of 20 years in which no countries eliminated malaria, between 2016 and 2019, Sri Lanka, Paraguay, Algeria, Argentina and Uzbekistan were certified as malaria-free with another 19 countries (the remaining “E2020” countries) identified with the potential to eliminate indigenous transmission by the year 2020 [3]. Despite significant progress in controlling and eliminating malaria, a recent increase in the global burden of malaria was recorded, with eleven countries (10 in sub-Saharan Africa plus India, the “10 + 1” countries) responsible for 70% of malaria cases globally [4].

Presently, the World Health Organization (WHO) recommended malaria vector control strategies for programmatic implementation are limited to ITNs and IRS against adult mosquitoes with larval source management (LSM) as a supplemental intervention [5]. All three strategies require insecticides. Since 2004, more than 2 billion pyrethroid-treated long-lasting insecticide-treated nets (LLINs) were distributed [1, 6]. While the number of LLINs distributed and in use continues to increase, the number of people protected by IRS, while still significant, diminished from 180 million globally in 2010 to 116 million in 2017 [1]. Significant unintended but inevitable challenges emerged from insecticide selection pressure from malaria control: vector distributions have changed [7, 8], insecticide resistance (IR) has grown [9] and biting patterns (locations and times) have shifted [10].

Despite these challenges, vector control interventions have one of the highest returns on investment in public health [11]. The WHO recommends universal coverage of LLINs or IRS for all populations at risk regardless of the vector species present or intensity of transmission. There are 41 dominant malaria vector species, each with unique behaviours presenting unique challenges for vector control [12]. Thus, the capacity of vectors to transmit malaria and their susceptibility to each vector control strategy will vary by species, location and epidemiological scenario. Further gains in controlling malaria require preserving the effectiveness of the presently recommended vector control interventions while integrating new control strategies based on the susceptibility of local vectors to specific interventions [13].

Recognizing this need, the Global Technical Strategy for Malaria 2016–2030 [14] transformed malaria surveillance, including entomological surveillance, into a core intervention in all countries vulnerable and receptive to malaria, with detailed guidance on prioritizing vector indicators in *Malaria Surveillance, Monitoring and Evaluation: A Reference Manual* [15]. This recommendation encompasses both malaria-endemic countries as well as countries that have eliminated malaria but remain susceptible to re-establishment of transmission. The WHO identified 5 main objectives of vector surveillance: (1) to characterize receptivity (for selection and stratification of interventions), (2) to track malaria vector densities (for timing of vector control deployment by seasonality of transmission), (3) to monitor IR (for choosing insecticides), (4) to identify other threats to vector control efficacy and (5) to identify gaps in vector control intervention coverage [15].

Monitoring these objectives requires tracking eight specific vector indicators (i.e., vector identification, vector density, blood feeding habits, indoor/outdoor biting, indoor/outdoor resting, IR, malaria infections and larval habitats). These activities and objectives are prioritized by the WHO by transmission intensity and the vector control interventions in use [15]. Country programmes should have a national strategic plan with a monitoring and evaluation framework that includes strategies for IR management, integrating control strategies and the use of vector data in programmatic decisions. Implementing activities to support the strategies detailed in the national framework requires an adequate capacity (i.e., manpower, infrastructure, transportation, budget). This capacity needs to be able to monitor vector indicators sufficiently frequently to identify trends that may negatively impact intervention effectiveness. Rapid detection of adverse trends will enable more proactive actions to maintain vector intervention efficacy in countries controlling malaria and to make controlling and eliminating countries resilient to respond rapidly to outbreaks.

To assess the current status of country malaria vector surveillance programmes, an online survey gathered fundamental metadata on the relationships between surveillance and intervention deployment, national vector control strategies and data collection techniques and how these data are used in making programmatic decisions. This needs assessment documented the types of vector indicator data being monitored by countries. The analyses that follows creates a baseline for defining the present status on surveillance activities against the recommended best practices by transmission intensity. Regional strengths and limitations in surveillance among the countries in Africa and the Asia-Pacific are identified and gaps between activities, strategies and programme management decisions are examined. This survey documented the vector surveillance and control activities conducted or coordinated by the National Malaria Control Programmes (NMCPs), and did not assess the rate of application or use of interventions and other tools.

## Methods

### Data collection

An online survey instrument was designed to capture descriptive information about activities conducted by the NMCPs, from the types of interventions deployed to vector surveillance activities and how vector data was used for decision-making. The survey was refined in consultation with the Asian Pacific Malaria Elimination Network, the African Leaders Malaria Alliance, the E8 Secretariat, the Malaria Consortium, the University of Notre Dame and the University of California-San Francisco Malaria Elimination Initiative. NMCPs and key partner organizations were approached to participate in the survey, both individually and through regional support networks. Information was collected on the metadata for control and surveillance activities conducted or coordinated through NMCPs (e.g., the percentage of countries that used an indicator and not the actual data describing the level of access and use of an intervention).

Drop down menus allowed informants to rapidly report (1) vector control interventions in use (both the WHO recommended strategies as well as personal and community control measures for which the evidence base is less well defined), (2) strategies of the national programmes (e.g., IR management, integrated vector control, use of data in decision-making), (3) vector surveillance indicators monitored, (4) techniques to measure vector indicators, (5) data management tools to record and present data and (6) how intervention access and use is monitored. Limitations to vector surveillance at the country programme level were self-identified by open-ended questions (analyses of surveillance limitations will be presented in a companion paper). To minimize mis-interpretation of country activities, many questions used drop-down menus, the survey was available in English, French and Spanish and clarifications to open-ended questions were sought via follow up emails. The survey is available at https://ee.kobotoolbox.org/x/#YNbC.

### Data analyses

Vector surveillance programmes were assessed against five major objectives for malaria vector surveillance programmes: (1) characterization of receptivity to guide stratification and selection of interventions, (2) tracking the relative density of malaria vector species to determine the seasonality of transmission and the optimal timing of interventions, (3) monitoring IR for choosing insecticide formulations, (4) identifying other threats to the effectiveness of vector control, and (5) monitoring vector control intervention coverage and quality to identify intervention coverage gaps and opportunities for other interventions [15].

Country vector surveillance programmes in Africa and Asia-Pacific were compared by region and by transmission intensity (control or eliminating) status using the metadata for WHO indicators. The proportion of NMCPs implementing recommended WHO interventions (ITNs, IRS and LSM) as well as other personal and programmatic interventions were analysed with a chi-squared test of proportions (*prop.test*). Questions with multi-categorical answers were compared between countries in Africa and Asia-Pacific using a Chi squared contingency table (*chisq.test*) (e.g., indicators to monitor ITN usage and coverage, survey types to monitor usage and coverage, and organizations coordinating surveys to monitor ITN usage and coverage). Comparisons of vector interventions deployed and national vector strategies in place between control and eliminating countries were analysed by 2-sample test for equality of proportions with continuity correction. These analyses were performed using the R package (v3.5.1).

## Results

### Global analyses of malaria vector control and surveillance by regions

#### Vector control interventions implemented

**Fig. 1:**
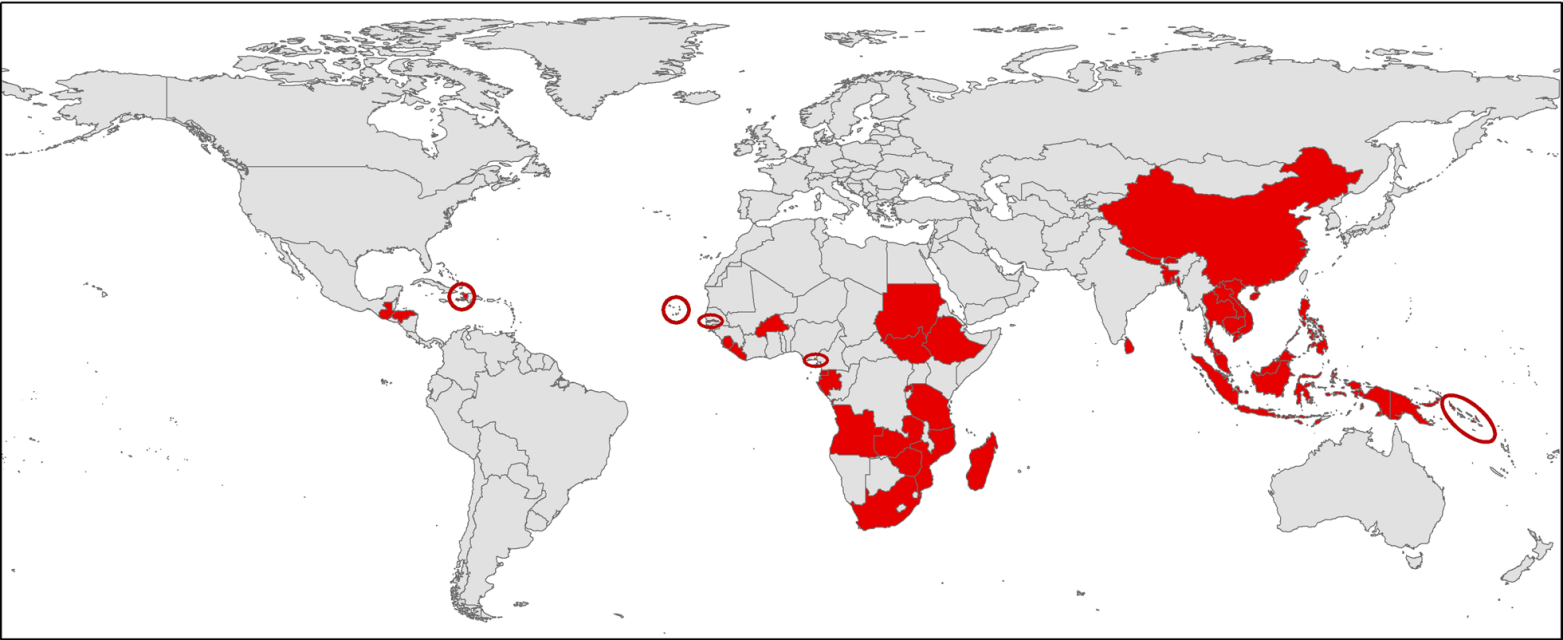
Distribution of the 35 malaria endemic countries that participated in the survey

Thirty-four malaria endemic countries and one country recently certified as malaria-free participated in the survey [Africa (n = 18), Asia–Pacific (n = 14) and the Americas (n = 3)] between 1 November 2017 and 19 November 2018 (Fig. 1). Most surveys were completed by an individual for each country and the responses reported may have varied if completed by a different individual or by a group.

**Fig. 2:**
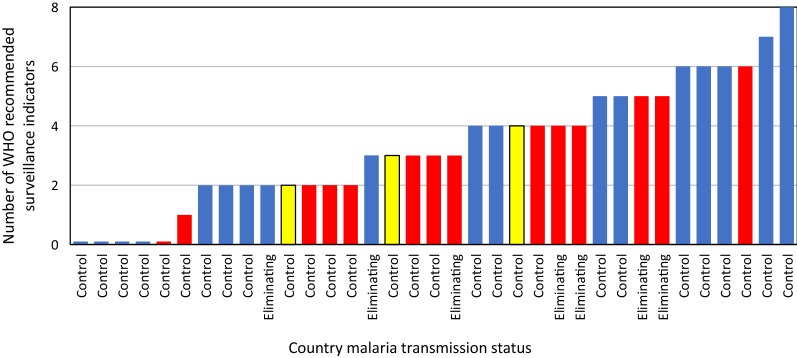
Number of WHO recommended indicators monitored by National Malaria Control Programmes (NMCP Africa countries shown in blue, Asia-Pacific in red and Americas in yellow). The 8 recommended indicators were: identifying vectors, quantifying vector densities by species, determining blood feeding habits, assaying indoor/outdoor biting and resting rates, determining insecticide resistance, tracking mosquito infection rates and surveying larval habitats [15]

Data on active monitoring by NMCPs of the eight WHO recommended priority vector indicators [15] were documented (Fig. 2). The number of vector indicators that programmes monitored ranged from none to all eight with 86% of country NMCPs monitoring at least one indicator. Due to the limited number of participating countries in the Americas. Regional analyses focused on country programmes in Africa and the Asia-Pacific where 97% of malaria cases occurred in 2017 [1].

**Fig. 3:**
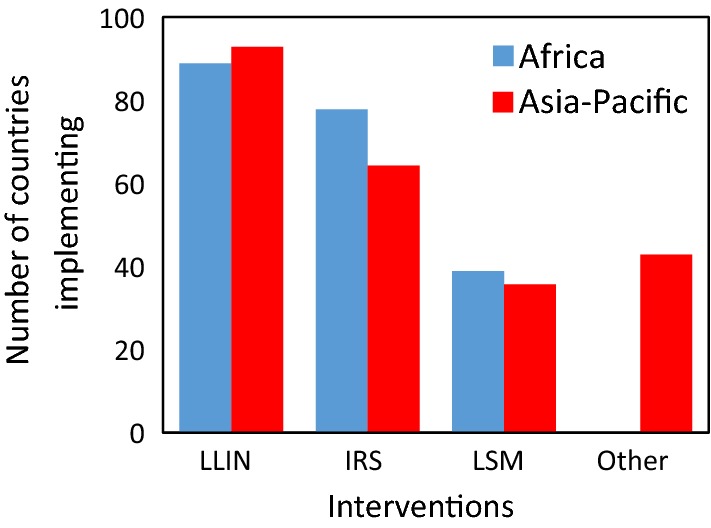
Vector control interventions deployed by National Malaria Control Programmes in the Africa and Asia-Pacific regions

Overall, 91% of participating countries distribute LLINs, 31% implement IRS and 41% practice LSM as part of the NMCP strategies (Fig. 3). Integrated vector control was part of the national strategy in 58% of countries, most frequently manifested as sympatric distributions of LLINs with IRS in areas with higher malaria rates. Statistical differences in vector intervention use by countries in the Africa and Asia-Pacific regions were not found (χ^2^ = 2.078, df = 3, p = 0.556).

Long-lasting insecticide-treated nets (LLINs) were distributed through focal responses and/or mass campaigns in the surveyed countries. Insecticides used in IRS differed significantly between the regions (χ^2^ = 11.51, df = 3, p = 0.009). In Africa, all four main insecticide classes were widely used [64% of countries used organophosphates, followed by pyrethroids (50%), carbamates (43%), and the organochlorine, DDT (21%)]. Pyrethroids were used in IRS by 100% of Asia-Pacific responding countries implementing IRS. Regional differences in LSM methods were not found (χ^2^ = 2.719, df = 3, p = 0.437) with larviciding being the dominant strategy, usually with an organophosphate (e.g., Temephos).

Other vector control methods were less frequently deployed (in 28% of countries) (Fig. 3). There was a greater tendency for NMCPs of the Asia-Pacific countries to incorporate other vector control interventions (Africa = 17%, Asia-Pacific = 43%; χ^2^ = 1.302, df = 1, p = 0.254) (e.g., outdoor space spraying, topical repellents, hammock nets, coils and mosquito proof housing).

#### Intervention use and coverage indicators

**Fig. 4:**
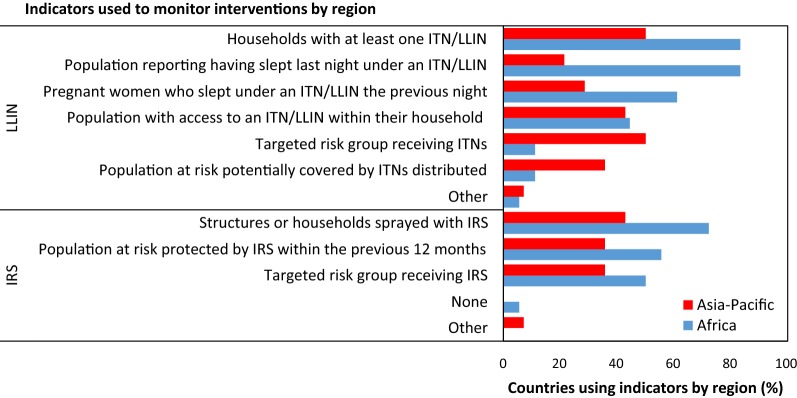
Indicators to monitor intervention coverage and/or use in countries by National Malaria Control Programmes in the Africa and Asia-Pacific regions

Regional differences in ITN usage and coverage indicators were found (χ^2^ = 14.29, df = 6, p = 0.026; Fig. 4). In Africa, 94% of responding countries used the indicators “the proportion of the population having slept the previous night under an ITN/LLIN”), and “the proportion of households with at least one ITN/LLIN”. In the Asia-Pacific there was more diversity in indicators used: the proportion of households with at least one ITN/LLIN and the proportion of targeted risk groups receiving ITNs was used in 54% of countries while the proportion of the population with access to an ITN/LLIN within their household was used in 46% of countries.

Regional differences in IRS coverage indicators were not found (χ^2^ = 2.53, df = 4, p = 0.639; Fig. 4); the proportion of structures or households sprayed with IRS was the most commonly used indicator of IRS coverage (Africa = 93%, Asia-Pacific = 67%). Other common indicators were the proportion of the population at risk protected by IRS (Africa = 71%, Asia-Pacific = 56%) and the proportion of target risk groups receiving IRS (Africa = 64%, Asia-Pacific = 56%).

Monitoring of LSM was undertaken by 75% of countries, usually as follow up surveys for the presence of larvae in treated habitats with survey frequency dependant on available resources.

**Fig. 5:**
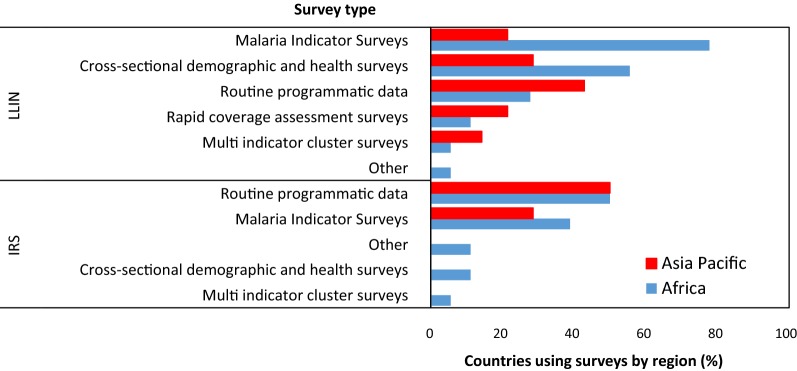
Surveys to monitor intervention coverage and/or usage by National Malaria Control Programmes in the Africa and Asia-Pacific regions

Types of surveys to monitor LLIN usage and coverage or IRS coverage did not differ between Africa and Asia-Pacific countries (LLIN: χ^2^ = 8.89, df = 5, p = 0.113; IRS: χ^2^ = 3.262, df = 4, p = 0.515; Fig. 5). For LLIN use and coverage, countries in Africa tended to use the Malaria Indicator Survey (88%) or cross-sectional demographic and health surveys (63%), while Asian-Pacific countries used data collected as routine programme data (46%). For IRS, countries in both regions favoured routine programmatic data to determine coverage (Africa = 64%, Asia-Pacific = 78%).

Statistical differences in the survey frequency to monitor ITN use and coverage or IRS coverage between African and Asian-Pacific countries were not found (LLIN: χ^2^ = 5.53, df = 5, p = 0.35; Additional file 1: Figure S1; IRS: χ^2^ = 8.921, df = 6, p = 0.178; Additional file 1: Figure S2). Annual surveys for ITN and IRS coverage were most frequently conducted (ITN: Africa = 33%, Asia-Pacific = 31%; IRS: Africa = 62%, Asia-Pacific = 38%).

Most countries distributing LLINs assessed physical durability (Africa = 80%, Asia-Pacific = 69%) and the effective life of the nets (Africa = 60%, Asia-Pacific = 85%). The proportion of countries assessing durability and effective life did not vary significantly between Africa and Asia-Pacific countries (physical durability: χ^2^ = 0.05, df = 1, p = 0.826; effective life: χ^2^ = 1.04, df = 1, p = 0.308).

Most countries conducting IRS assessed the initial efficacy (Africa = 86%, Asia-Pacific = 89%) and the effective life (Africa = 93%, Asia-Pacific = 100%) of insecticides using cone tests or wall bioassays. Significant differences between the Africa and Asia-Pacific countries in the proportion of countries assessing IRS efficacy were not found (initial efficacy: χ^2^ < 0.001, df = 1, p = 0.99; effective life: χ^2^ < 0.001, df = 1, p = 0.99).

### Routine surveillance of vector indicators

**Fig. 6:**
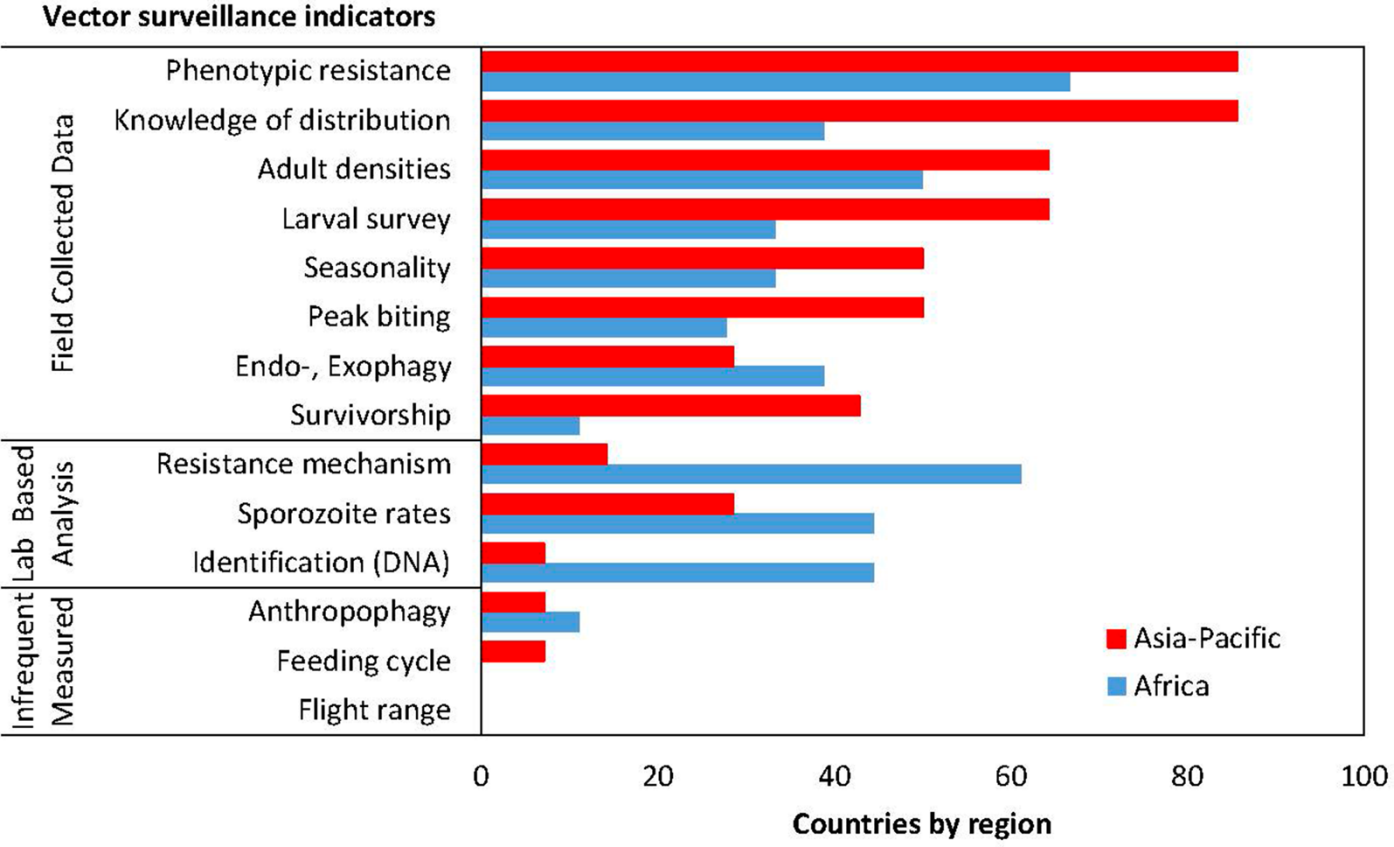
Vector surveillance indicators monitored by countries in the Africa and Asia-Pacific regions

Routine vector surveillance, of at least one indicator, is conducted annually in 74% of countries, with no difference between the Africa and Asia-Pacific regions (χ^2^ = 0.008, df = 1, p = 0.925). The range of vector surveillance indicators monitored is presented in Fig. 6.

#### Vector density and behaviours

**Fig. 7:**
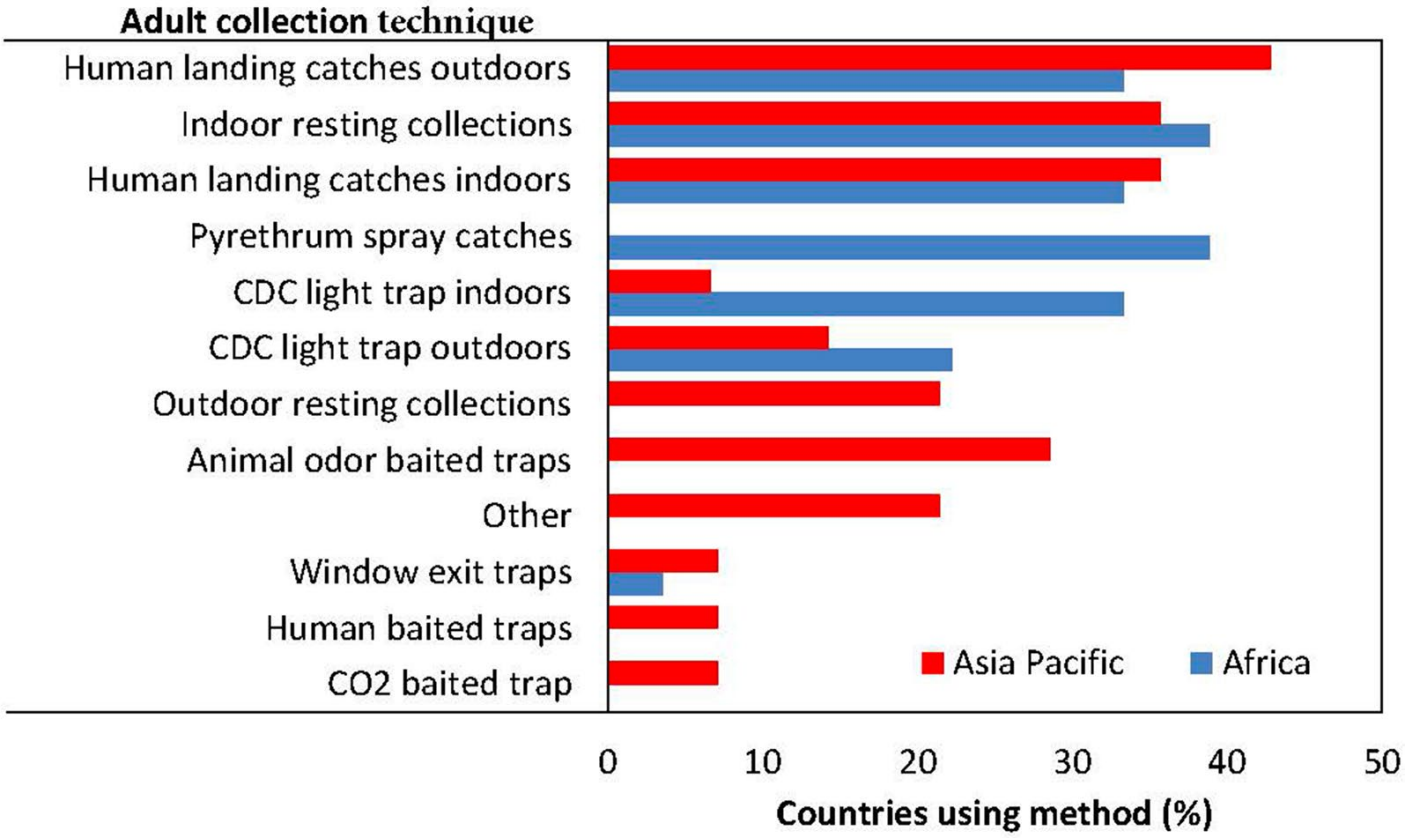
Techniques to monitor adult vector densities in Africa and Asian-Pacific countries

Monitoring adult densities is a fundamental surveillance activity needed to determine seasonality, indoor/outdoor biting, peak biting time and to map spatial distributions. Post-collection analysis is required to determine three indicators: sporozoite rates, survivorship, and human blood indices. Fifty-six per cent of countries measured adult vector densities at least once a year, with no difference between the Africa and Asia-Pacific regions (χ^2^ = 0.074, df = 1, p = 0.786). The main adult collection techniques were human landing catches (indoors or outdoors) and indoor resting collections in both regions (Fig. 7). Adult densities were measured frequently enough to determine seasonality in 41% of countries, with no difference between the Africa and Asia-Pacific regions (χ^2^ = 0.211, df = 1, p = 0.645). Peak biting times were measured in 38% of countries; and was more frequently measured in the Asia-Pacific (50%) compared with countries in Africa (28%), but this difference was not significantly different (χ^2^ = 0.846, df = 1, p = 0.357). Indoor/outdoor biting rates were determined in 34% of countries, with no difference between the Africa and Asia-Pacific regions (χ^2^ = 0.054, df = 1, p = 0.814). The sporozoite rate was measured in 38% of countries, with no difference between the Africa and Asia-Pacific regions (χ^2^ = 305, df = 1, p = 0.581).

Larval surveys were conducted at least annually in 47% of countries [no difference between the Africa and Asia–Pacific regions (χ^2^ = 1.914, df = 1, p = 0.166)].

#### Vector distributions

**Fig. 8:**
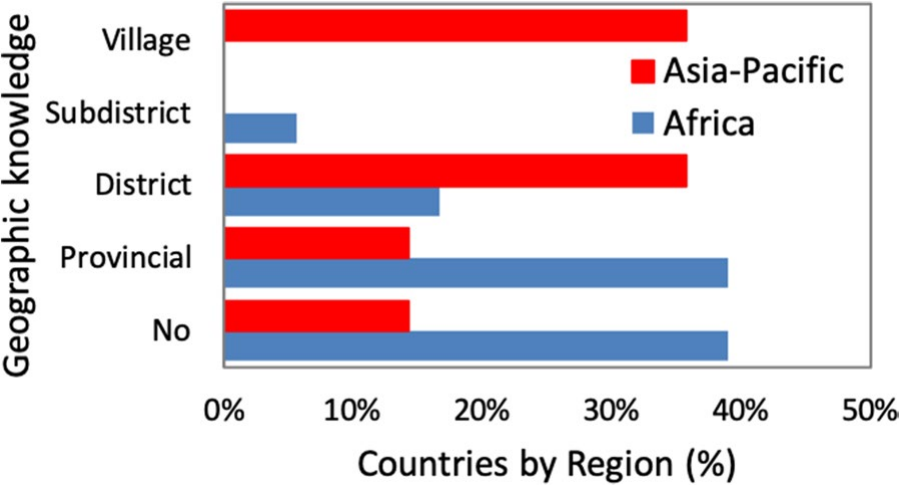
Knowledge of the geographic distribution of malaria vector species in Africa and Asian-Pacific countries

Eighty-six percent of countries in the Asia-Pacific knew the distributions of their vectors compared with 61% of countries in Africa. The geographic scale at which country vector distributions were known differed significantly between the Africa and Asia-Pacific regions (χ^2^ = 11.73, df = 4, p = 0.019; Fig. 8). Asia-Pacific countries knew their vector distributions at a finer scale (36% to the village level) compared to countries in Africa. However, the methods of identifying vector species differed between the regions (χ^2^ = 6.4, df = 1, p = 0.011). Morphology only based identifications of mosquitoes was used in 92% of countries in the Asia-Pacific, while 67% of countries in Africa confirm morphological identifications with molecular techniques.

#### Insecticide resistance

##### Phenotypes

Insecticide resistance phenotypes of adult vectors was measured, at least annually, in 75% of countries (Africa = 67%, Asia-Pacific = 86%;) (Fig. 6). While there was no difference between the proportion of countries by regions measuring phenotypic insecticide resistance (χ^2^ = 0.149, df = 1, p = 0.698), the regions differed significantly in the methods to collect specimens for resistance testing (χ^2^ = 15.38, df = 2, p = 0.0004). In Africa, anopheline adults reared from larvae were used 75% of the time, while in the Asia-Pacific wild caught adults were used by 82% of respondents. Over half of the countries (58%) pooled all wild caught *Anopheles* species for testing (no differences between the regions; χ^2^ = 0.078, df = 1, p = 0.779).

Of the countries monitoring phenotypic resistance, all (100%) countries monitored adult vectors with the WHO tube test. While all of these countries monitored for pyrethroid resistance, fewer countries also monitored resistance to organophosphates, carbamates and organochlorines with the WHO tube test. The CDC bottle bioassay was also used to characterize resistance phenotypes in a few countries in each region (Africa = 20%, Asia-Pacific = 8%). Insecticide resistance surveillance sites in each country ranged from 1 to 30, with testing frequency between quarterly and annually. Only one country (in the Asia-Pacific) routinely tested for resistance in larvae (against temephos).

Of the 29 countries distributing LLINs, 76% monitored for pyrethroid resistance but none evaluated vector for resistance to pyrethroids with the synergist, piperonyl butoxide (PBO). Of the countries that used pyrethroids in IRS (n = 16), 88% monitored pyrethroid resistance. All countries that used organophosphates (n = 9), evaluated vectors for organophosphate resistance while 86% of countries (n = 7) using carbamates in IRS monitored for carbamate resistance but only one of three countries (n = 3) using the organochlorine, DDT, in IRS monitored resistance to DDT.

##### Mechanisms

Regional differences in molecular testing for resistance mechanisms were found with 61% of countries in Africa, but only 14% of Asia-Pacific countries, evaluating insecticide resistance mechanisms (χ^2^ = 5.348, df = 1, p = 0.021). In African countries, the most common resistance mechanisms tested were L1014S and kdr L1014F knock down resistance (kdr) (Additional file 1: Figure S3). In the Asia-Pacific, mechanism testing was primarily for esterases (kdr presence was not tested).

### Other vector indicators

Mosquitoes survivorship was determined using parity dissections in 25% of countries, with no difference between the Africa and Asia-Pacific regions (χ^2^ = 2.709, df = 1, p = 0.099) (Fig. 6). The feeding cycle length was only measured by one country in the Asia-Pacific. Anthropophagy was only monitored in 9% of countries: 2 African countries and 1 Asia-Pacific country. The flight range of vectors was not measured in any of the surveyed countries.

### Analyses of vector interventions and strategies by control status

#### Vector control interventions and strategies by country transmission intensity

Of the 35 participating countries, six were classified as “eliminating” based on their inclusion in the E2020 and one (Sri Lanka) was certified as malaria-free in 2016 (Table 1, Fig. 2). The remaining 28 countries were in the malaria control phase. Interventions deployed and management strategies were compared between eliminating and control countries at a global scale due to the small number of participating eliminating/eliminated countries. Large differences were noted in the proportion of control versus eliminating countries using various vector control strategies. While all eliminating countries implemented IRS, only 57% of eliminating countries distributed LLINs. Conversely, all countries in the control phase distributed LLINs but only 61% included IRS in their national malaria control strategies. The difference in the proportion of control versus eliminating countries deploying LLINs was statistically significant (χ^2^ = 8.226, df = 1, p = 0.004). Larval source management was almost twice as likely to be reported being used in eliminating compared to countries controlling malaria while non-recommended interventions were more than threefold more likely to be used in eliminating countries and this difference was statistically significant for the alternative (non-WHO recommended) interventions (χ^2^ = 5.469, df = 1, p = 0.019).

**Table 1:** Proportion of National Malaria Control Programme (NMCP) implementing vector control interventions and strategies by region and control status

Region	Malaria status	Interventions deployed				Vector control strategies		
		IRS	ITNs	LSM	Other	IRM	Integrated Control	Use data
Asia-Pacific	Eliminating (n = 5)	1.00	0.80	0.60	0.60	0.20	0.80	0.80
	Control (n = 9)	0.44	1.00	0.22	0.33	0.44	0.33	0.78
Africa	Eliminating (n = 2)	1.00	0.00	1.00	1.00	0.50	1.00	1.00
	Control (n = 16)	0.75	1.00	0.31	0.06	0.67	0.60	0.50
Americas	Control (n = 3)	0.33	1.00	1.00	0.33	0.33	1.00	0.33
Global	Eliminating (n = 7)	1.00	0.57*	0.71	0.71**	0.29	0.86	0.86
	Control (n = 28)	0.61	1.00*	0.36	0.18**	0.54	0.60	0.58
	Overall (n = 35)	0.69	0.91	0.43	0.29	0.49	0.66	0.64

* Proportions differ significantly by chi-squared test of proportions, p = 0.004

** Proportions differ significantly by chi-squared test of proportions, p = 0.019

Significant differences between the proportion of countries in the control and elimination phases that had strategies for IR and integrating multiple vector control strategies or that used vector data as part of their decision-making process were not found, although IR management strategies were more commonly reported in control compared to elimination countries (54% and 29%, respectively). Despite the WHO call for countries to have IR management plans and for implementing integrated vector management, only 49% of countries reported having an IR management plan with 66% deploying multiple sympatric vector control strategies.

#### Vector data-based decision-making

Vector data was used in decision making by 64% of countries (Table 1). The most common techniques for storing data was using Excel in 70% (19 of 27) of responding countries or with paper-based methods as reported by 63% of countries (17 of 27 countries). Vector surveillance data was most frequently presented using Excel (38% of countries) followed by data dashboards (in 28% of countries). Spatial decision support systems were employed in 14% (3 of 21 countries).

Vector surveillance data informed seven categories of decisions (Table 2). The most commonly cited use for vector data was to stratify adult vector control strategies (24% of countries). The second and third most commonly identified roles for vector data was in selecting insecticides (18% of countries) and larval control strategies (12% of responding countries). Three countries each used data to define receptive areas and to identify sites for focus studies. While 69% of countries use IRS, data was used by 3% of countries to determine where IRS should be applied despite data on indoor/outdoor resting behaviours being collected in 34% of countries. Only one country used data on LLIN durability to select LLINs for distribution despite 91% of surveyed countries distributing ITNs as part of their national malaria control strategy. While 77% of countries monitor for IR, only 18% consider vector data when selecting insecticides for programmatic use. LSM is implemented in 43% of countries but only 12% select larval control strategies based on larval survey data (and only 3% of countries test for IR in larvae). Differences between eliminating and control countries in how data were used were not seen.

**Table 2:** Proportion of National Malaria Control Programmes (NMCPs) utilizing vector data in decision-making by region and control status

Region	Malaria status	Programmatic decisions						
		Insecticide selection	Resting location for IRS	Durability for LLIN selection	Larval control choice	Adult control strategies	Defining receptivity	Identify sites for focus
Asia-Pacific	Eliminating (n = 5)	0.00	0.00	0.00	0.00	0.40	0.20	0.20
	Control (n = 9)	0.00	0.00	0.00	0.22	0.44	0.22	0.11
Africa	Eliminating (n = 2)	0.50	0.50	0.00	1.00	0.00	0.00	0.50
	Control (n = 14)	0.36	0.07	0.07	0.00	0.07	0.00	0.00
Americas	Control (n = 3)	0.00	0.00	0.00	0.00	0.33	0.00	0.00
Global	Eliminating (n = 7)	0.14	0.00	0.00	0.29	0.29	0.14	0.14
	Control (n = 26)	0.19	0.04	0.04	0.08	0.23	0.08	0.04
	Overall (n = 33)	0.18	0.06	0.03	0.12	0.24	0.09	0.09

#### Vector surveillance by transmission intensity-based recommendations

The countries in the elimination phase monitored a mean of 4.7 vector surveillance indicators whereas countries in the control phase monitor a mean of 3.8 indicators (Table 3). The WHO guidelines stratify the importance of vector surveillance indicators by transmission intensity. In eliminating countries, it is recommended to track receptivity (a function of vector distributions and population size) and to define IR phenotypes. The seven eliminating countries monitor these three priority indicators with greater frequency than countries controlling malaria; for eliminating countries, 86% monitor IR phenotypes, 86% conduct larval surveys and 100% knew their vector distributions compared to 75%, 39% and 64% of countries controlling malaria, respectively. Of the six other prioritized vector surveillance indicators for high transmission countries, the only indicator that was measured in more than half of countries was annual adult densities (57%). For the remaining indicators, the frequency at which they were measured was 43% for IR mechanisms, 32% for sporozoite rates, indoor/outdoor biting and resting behaviours with only 11% measuring the human blood index annually.

**Table 3:**
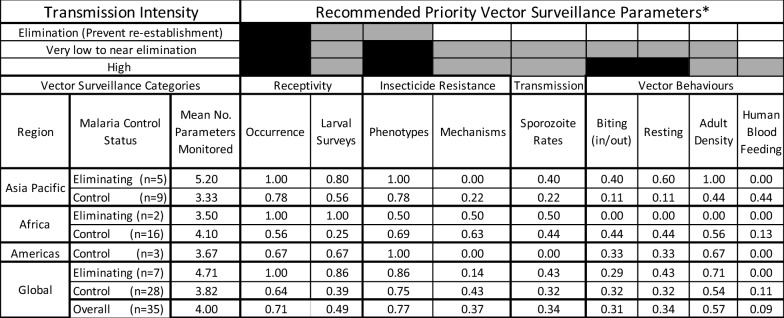
Proportion of National Malaria Control Programmes (NMCPs) monitoring the WHO prioritized vector surveillance indicators by region and control status Adapted from [15]

## Discussion

Vector surveillance is fundamental for providing critical data for decision-making to ensure that malaria control programmes are, and remain, effective despite increasing resistance to insecticides [16]. Furthermore, as new control strategies and products receive recommendations for programmatic implementation, selecting the most effective combinations of interventions for programme use will require routine surveillance data on vector behaviours.

These analyses identified differences by geographic regions (Africa and Asia-Pacific) and transmission intensity (countries in the elimination or control malaria phases) in vector surveillance and control activities by NMCPs as well as how vector data is used in making decisions on intervention deployment.

One of the greatest challenges faced by NMCPs today is the increasing development of insecticide resistance, expressed as either physiological or behavioural resistance [9, 17]. Given the limited number of WHO-recommended malaria vector control interventions, and their dependence on insecticides, the high prioritization of NMCPs to monitor insecticide susceptibility phenotypes is reassuring. There was high concordance between insecticides in programmatic use and active monitoring for resistance phenotypes of those insecticides; and this is an essential pre-requisite for maintaining intervention effectiveness by enabling more proactive management for IR [18]. However, while 76% of countries monitor for IR, only half of NMCPs had an IR management strategy and fewer than 1 in 5 reported using vector data to choose insecticides for malaria vector control. Several critical gaps were found: despite a high prevalence of countries monitoring IR phenotypes, not all countries monitoring IR have strategic frameworks for responding to the data (e.g., IR management plans) and among those with a management plan, not all report using their data to select insecticides for programme use.

Vectors can develop behavioural resistance by avoiding contact with insecticides [10, 19]. Behavioural resistance is expressed as changes in either the location where vectors seek blood meals (i.e. shifting from indoors to outdoors) or the time when blood meals are taken (i.e. feeding earlier in the evening or in the morning when people are outside their houses and not protected by ITNs or IRS). Most NMCPs are unlikely to detect the emergence of behavioural resistance with their existing vector surveillance programmes as only 31% of countries monitor the indoor–outdoor biting ratio with 34% tracking changes in peak biting time, the two most common expressions of behaviour resistance. Enhanced response to behavioural resistance will require implementation of LSM or must await the availability of a novel intervention against adult vectors that operates outside houses.

A second gap exists in countries with an integrated vector control strategy (two-thirds of countries reported practicing integrated control) in the use of vector surveillance data in the design and implementation of this strategy. While 89% of countries collected vector data on at least one of 8 priority vector indicators [15], only 64% of countries identified one or more programme decisions made with consideration of vector data. More frequent consideration of vector data already being collected, coupled with increasing the number of priority indicators monitored as well as the geographic scale and frequency at which indicators are monitored would provide a firmer evidence base for making decisions to maintain or improve the effectiveness of malaria vector control.

As countries transition from controlling to eliminating malaria, the priority recommended vector surveillance indicators are more limited in number, focusing on defining receptivity (knowing the distribution of vectors and using larval surveys to map distributions and population sizes) and monitoring IR phenotypes. The dynamic nature of vector population responses to effective intervention deployment is expressed as changes in vector species distributions and increases in IR. As a consequence, receptivity/vector distribution maps require continual updating for meaningful stratified intervention deployment and for insecticide selection as part of an IR management strategy.

The eliminating countries in this survey monitored each of the priority indicators at a high level (86% of countries conduct annual larval surveys and determine IR phenotypes while 100% know their vector distributions). However, the specificity of vector identifications based on morphology alone predominates in Asia-Pacific countries. Hence, vector receptivity is mostly defined in the Asia-Pacific on the presence of vector complexes. Where the complex of morphologically indistinguishable species includes both vectors and non-vectors, stratifying vector control becomes problematic with the potential to be either cost-ineffective (deploying control in non-receptive areas) or by not providing vector control to vulnerable populations in receptive areas.

While the status of vector surveillance is variable across the countries participating in the survey, a number of encouraging attributes of national malaria vector surveillance programmes were identified. These include the priority that countries made to monitor IR phenotypes and the concordance between insecticides used for malaria control and the resistance profiles monitored. Having an established baseline of insecticide susceptibility profiles coupled with current intervention coverage and use data will facilitate rapid responses to outbreaks.

## Conclusions

Overall, Asia-Pacific NMCPs monitored more field vector indicators while African NMCPs more frequently monitored indicators requiring laboratory analyses of field-collected mosquitoes. This may reflect the input of the President’s Malaria Initiative (PMI) which was first established in Africa in 2005 and more recently in the Greater Mekong Subregion in 2011 (https://www.pmi.gov/about). Country institutes and universities in both regions have both the manpower and infrastructure for laboratory analyses but the linkage between research institutions and control programmes may need strengthening to fill data gaps.

Although an increasing number of countries are eliminating malaria or been certified as having achieved elimination, the need for vector surveillance will not diminish. The threats of physiological and behavioural resistance to insecticides in recommended interventions are increasing and the danger of re-introduction of transmission in eliminated areas that are both receptive and vulnerable will grow as more counties achieve elimination. As novel control strategies with new active ingredients receive WHO recommendations, the need for current data on vector behaviours will increase to enable rational vector control strategy selection.

This survey established a baseline for defining the present status of surveillance for malaria vectors and thus provides a benchmark against which programmatic changes in surveillance can be compared. Programme weaknesses identified by NMCPs in the course of participating in this survey can focus efforts on the key indicators requiring improved monitoring. A well-established vector surveillance programme will enable programmes to transition from reacting to outbreaks and threats to intervention effectiveness to evidence-based and proactive to emerging threats.

## Supplementary information

**Additional file 1.** Graphical presentation of parameters comparing the African and Asia-Pacific regions.

## Data Availability

Summarized data is available upon request, but country specific data will remain confidential unless written authorization to release the data is granted by the specific national malaria control authorities. Aggregated data is available at 10.25903/5dba680ab310.

## Abbreviations

IR: insecticide resistance
IRS: indoor residual spraying
ITN: insecticide-treated nets
LLIN: long-lasting insecticide-treated net
LSM: larval source management
NMCP: National Malaria Control Programme
PMI: President’s Malaria Initiative
WHO: World Health Organization
