# Supplementary material for: A global analysis of National Malaria Control Programme vector surveillance by elimination and control status in 2018
Source: Malar J. 2019 Dec 4;18:399. doi: 10.1186/s12936-019-3041-2 (PMC6894334; doi:10.1186/s12936-019-3041-2)

**Additional file**

CAPTION: Graphical presentation of parameters comparing the African and Asia-Pacific regions

**Figure S1.** Frequency of surveys conducted to monitor LLIN usage and coverage. Note that each bar represents the number of positive respondents standardised the number of countries that responded in each region. Respondents could select only one choice.


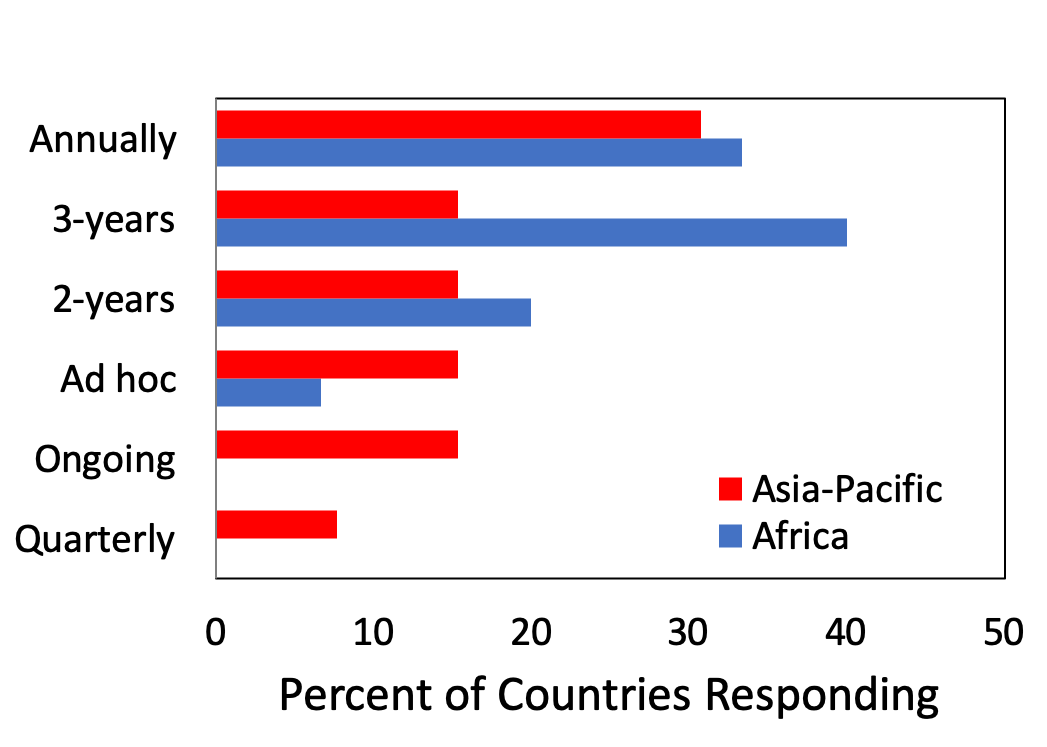


**Figure S2.** Frequency of surveys conducted to monitor IRS coverage. Note that each bar represents the number of positive respondents standardised the number of countries that responded in each region. Respondents could select only one choice.


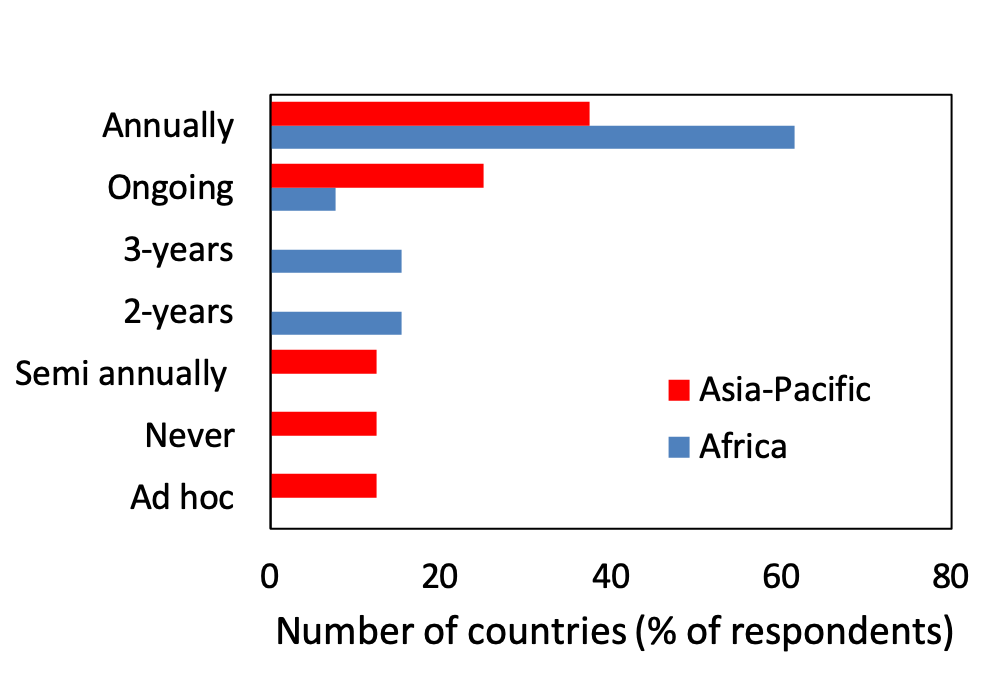


**Figure S3.** The types of resistance mechanisms that were tested for by countries in the African and Asia-Pacific regions. Note that each bar represents the number of positive respondents standardised the number of countries that responded in each region. Respondents were able to select more than one choice.


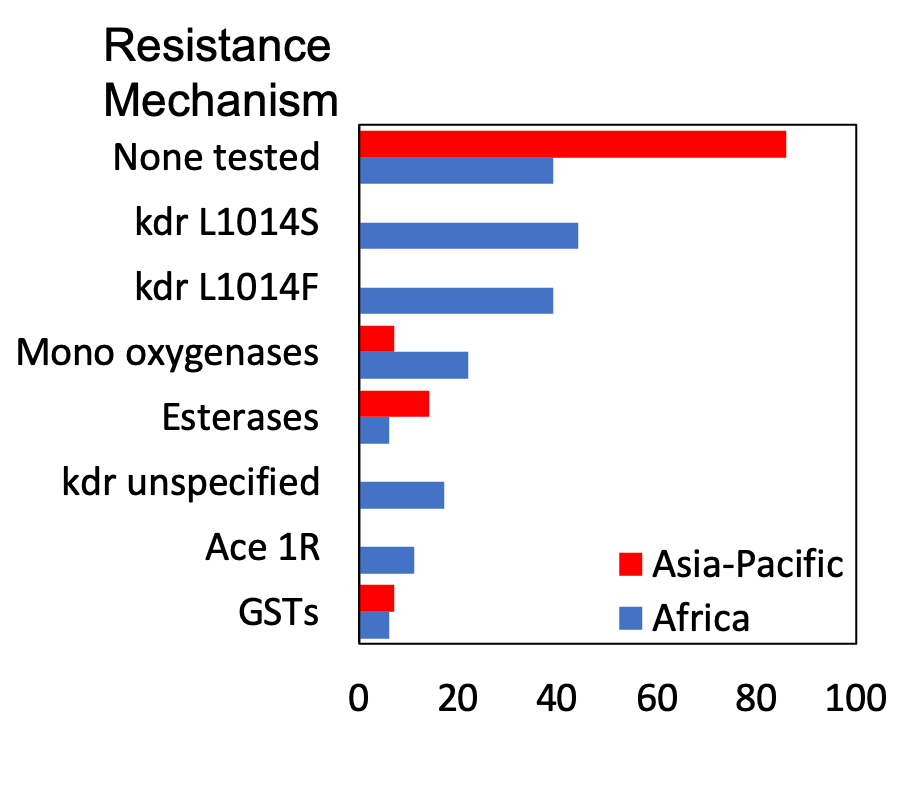

Supplement: Supplementary file 1 — Additional file 1. Graphical presentation of parameters comparing the African and Asia-Pacific regions. [file 12936_2019_3041_MOESM1_ESM.docx]
